# Cochrane corner: factors that influence compliance by healthcare workers with infection prevention and control guidelines for COVID-19 and other respiratory infections

**DOI:** 10.11604/pamj.supp.2020.35.2.23012

**Published:** 2020-05-06

**Authors:** Sara Cooper, Alison Wiyeh, Bey-Marrié Schmidt, Charles Shey Wiysonge

**Affiliations:** 1 Cochrane South Africa, South African Medical Research Council, Cape Town, South Africa; 2 School of Public Health and Family Medicine, Faculty of Health Sciences, University of Cape Town, South Africa; 3 Department of Epidemiology, University of Washington School of Public Health, Seattle, Washington, USA; 4 Department of Global Health, Faculty of Medicine and Health Sciences, Stellenbosch University, Cape Town, South Africa

**Keywords:** Coronavirus disease 2019 (COVID-19), Infection Prevention and Control (IPC), healthcare workers, cochrane review of qualitative evidence

## Abstract

As rates of novel coronavirus disease 2019 (COVID-19) continue rising in Africa, usage of infection prevention and control (IPC) strategies by healthcare workers (HCW) is critical. We highlight a Cochrane review of qualitative evidence that explored barriers and facilitators to HCW compliance with IPC recommendations for COVID-19 and other respiratory infectious diseases. The review found various individual- and organizational- level barriers and facilitators. The findings suggest that healthcare system constraints that make it difficult for healthcare workers to implement IPC guidelines require urgent prioritisation. This will help lay the foundation for addressing the more individual-level barriers potentially discouraging HCW from implementing IPC guidelines. We draw attention to pan-African initiatives for enhancing healthcare workers’ capacity to undertake IPC measures at such a critical time.

## Commentary

In this commentary we discuss the findings of a rapid Cochrane review of qualitative research that explored the barriers and facilitators to healthcare workers´ compliance with infection prevention and control (IPC) recommendations for respiratory infectious diseases [1]. We focus this article on the significance of the review and implementation considerations for African countries as they respond to the novel coronavirus disease 2019 (COVID-19) pandemic. COVID-19 has spread rapidly around the world since the first case was reported in China in December 2019. While the disease was initially slow to reach African countries compared to other countries, infections are currently rising exponentially on the continent and are likely to cause severe illness and death [2]. Based on the available evidence, the novel coronavirus is transmitted between people through close contact and droplets, and therefore those most at risk of infection are people who are in close contact with, or caring for, COVID-19 patients [3]. Within this context, rigorous usage of IPC strategies amongst healthcare workers is critical to protect themselves and prevent transmission in healthcare settings. Such strategies include, for example, the use of personal protective equipment (PPE), isolating infected patients from other patients and visitors, and stricter hygiene procedures within healthcare facilities [3]. A better understanding of the facilitators and challenges healthcare workers face in implementing IPC guidelines will help us devise evidence-informed strategies to enhance enablers and overcome barriers. Such strategies are essential and urgent as the virus begins to take its toll on the continent.

The Cochrane review by Houghton and colleagues [1] synthesized qualitative studies exploring the experiences and perceptions of healthcare workers regarding the factors that impact on their ability to comply with IPC recommendations for respiratory infectious diseases. The review included studies that focused on acute respiratory IPC guidelines or recommendations (local, national or international) in any healthcare setting, including primary care, acute hospital, long-term care, or community settings. Respiratory infectious diseases included in the review consisted of acute respiratory infections, COVID-19, severe acute respiratory syndrome (SARS), Middle East respiratory syndrome, tuberculosis, and influenza-like illness. Understandings of IPC for respiratory infections were guided by World Health Organisation (WHO) definitions [4]. Qualitative and mixed methods studies from any setting globally and published in any language after 2002 (in light of the SARS outbreak that occurred in 2003) were included.

A comprehensive search up to 26 March 2020 was conducted in Ovid MEDLINE, together with hand-searching of study reference lists. Subsequent screening of the search outputs and study selection produced 36 eligible studies; from which 20 studies were purposively sampled for inclusion in the synthesis. The five-stage ´best fit´ a priori framework synthesis approach was used to analyse and synthesise findings. The ‘Theoretical Model to Explain Self-Protection Behaviour at Work’ (PRECEDE) model was used as the guiding thematic framework. This model organises factors related to self-protective behaviour at work into three categories: individual (e.g. beliefs, attitudes, values), environmental (e.g. equipment availability and physical environmental factors), and organisational (e.g. training and education, policies and management expectations) factors. Methodological limitations were assessed with an adapted version of the Critical Appraisal Skills Programme (CASP) assessment tool. Confidence in the review findings was evaluated using the GRADE Confidence in the Evidence from Reviews of Qualitative Research (CERQual) approach. GRADE-CERQual categorizes confidence in the evidence from high (it is highly likely that the review finding is a reasonable representation of the phenomenon of interest) to very low (it is not clear whether the review finding is a reasonable representation of the phenomenon of interest). A range of factors were found to influence healthcare workers´ capability and motivation to follow IPC guidelines, with barriers and enablers frequently mirroring each other. All factors highlighted here were based on findings assessed as being of moderate to high confidence.

Barriers related to the guideline itself and how it is communicated include lengthy, poorly communicated and frequently changing guidelines that are inconsistent with broader national or international recommendations. Key factors motivating healthcare workers to adhere to IPC guidelines comprise fear of infecting themselves or others, feelings of professional responsibility for effective control practices, and a sense of the value in and importance of the guidelines. The physical discomfort of using masks and other equipment and the enhanced workloads and fatigue from implementing IPC strategies served as discouraging factors for healthcare workers´ application of IPC procedures. Healthcare workers were demotivated to use PPE if the equipment would make patients feel isolated, frightened, or stigmatised. Healthcare workers reported that their responses to IPC guidelines were influenced by the degree of support they felt they received from their management team, the workplace culture and influence of colleagues (e.g. culture of complacency, or social norm of wearing PPE) and whether there was mandatory and adequate training about the infection itself and how to use PPE. Several resource-related factors associated with access, availability and the physical environment were identified as critical to enabling the implementation of IPC guidelines. These include sufficient space to isolate patients (e.g. isolation rooms, anterooms and shower facilities), provision of good ventilation, adequate supply of PPE and other supplies of sufficient quality, and healthcare facilities where overcrowding and visitations are minimised, infected patients can be fast tracked and isolated, and handwashing facilities and surface decontamination supplies are available and easily accessible.

## Conclusion

The findings from this review highlight the importance of involving and empowering all facility healthcare workers and support staff as primary partners when implementing IPC guidelines. Twelve of the sampled studies were from high-income countries, and studies from only two African countries (three studies from South Africa and one study from Uganda) were included in the analysis. As experiences and perceptions of healthcare workers are potentially context-specific, the findings of this review need to be interpreted for African countries with some degree of caution. While more qualitative research on this topic is needed in African countries, the findings from this review have various policy and practice implications for responding to the COVID-19 pandemic on the continent. The healthcare system constraints that make it difficult for healthcare workers to implement IPC guidelines require urgent attention and prioritisation. This will help lay the foundation for addressing the more individual-level barriers that may demotivate healthcare workers from implementing IPC guidelines for COVID-19 in Africa.

Currently, the global shortage of appropriate PPE, such as surgical masks, N95 respirators, gowns, and goggles for front-line healthcare workers [3] as well as the surging numbers of coronavirus infections have overwhelmed even highly resourced healthcare systems [5]. These challenges are likely to be amplified in Africa, where many healthcare systems are plagued by limited and inequitable funding, supply scarcities, and an overstretched and inadequately supported workforce that receives insufficient in-service training [6]. Thus, significant investments and dedicated resources are urgently required to ensure healthcare facilities have the equipment, supplies, and amenities necessary for COVID-19 infection prevention and control activities. Moreover, global recommendations for optimizing the availability of PPE for healthcare workers [3] should be drawn upon and appropriately tailored to local contexts. For example, infrastructure and workforce teams could be reorganised and repurposed in innovative ways to ensure that the physical environment of healthcare facilities is conducive for IPC activities. Here the wealth of experience and valuable insights that many African countries have gained from dealing with previous and ongoing disease outbreaks could be utilised or adapted [7].

At the same time, training and education interventions which impart knowledge about the virus and equip healthcare workers with skills on how to use PPE and implement other IPC guidelines are required. In an overview of systematic reviews, several skills building strategies for implementation in health systems in low- and middle-income settings were found to have varying levels of effectiveness, including practice facilitation, educational outreach, audit and feedback, educational meetings, and local opinion leaders [8]. These could be drawn upon to inform the development and implementation of COVID-19 infection prevention and control initiatives in African countries. There are positive signs that these kinds of initiatives are emerging on the continent [9]. For example, the Africa Centres for Disease Control and Prevention (Africa CDC) has taken the initiative to co-ordinate regional and regional and international partners to promote evidence-based public health responses to COVID-19 [10]. ´IPC in healthcare facilities´ and ´supply chain and stockpiling´ constitute two of the six technical focal areas. The former will concentrate on organising and facilitating training for national IPC focal persons, providing on-site technical assistance for the development and implementation of protocols in healthcare facilities of Member States, and developing and updating “readily comprehensible, practical IPC guidance, based on evolving evidence and guidance from WHO” [10]. In terms of supply chain and stockpiling, the strategy will focus on building relationships with reliable manufacturers and managing supply chains for shared continental resources, including PPE. More of these kinds of pan-African initiatives, which strengthen communication, pool resources and consolidate efforts within the continent, are needed if we hope to provide healthcare workers with the resources and expertise required to undertake IPC measures at such a critical time.

## Competing interests

The authors declare no competing interests.
